# Visualisation of abscisic acid and 12-oxo-phytodienoic acid in immature *Phaseolus vulgaris* L. seeds using desorption electrospray ionisation-imaging mass spectrometry

**DOI:** 10.1038/srep42977

**Published:** 2017-02-17

**Authors:** Hirofumi Enomoto, Takuya Sensu, Kei Sato, Futoshi Sato, Thanai Paxton, Emi Yumoto, Koji Miyamoto, Masashi Asahina, Takao Yokota, Hisakazu Yamane

**Affiliations:** 1 Department of Biosciences, Faculty of Science and Engineering, Teikyo University, Utsunomiya 320-8551, Japan; 2 Division of Integrated Science and Engineering, Graduate School of Science and Engineering, Teikyo University, Utsunomiya 320-8551, Japan; 3 Waters Corporation, Shinagawa 140-0001, Japan

## Abstract

The plant hormone abscisic acid (ABA) and the jasmonic acid related-compound 12-oxo-phytodienoic acid (OPDA) play crucial roles in seed development, dormancy, and germination. However, a lack of suitable techniques for visualising plant hormones has restricted the investigation of their biological mechanisms. In the present study, desorption electrospray ionisation-imaging mass spectrometry (DESI-IMS), a powerful tool for visualising metabolites in biological tissues, was used to visualise ABA and OPDA in immature *Phaseolus vulgaris* L. seed sections. The mass spectra, peak values and chemical formulae obtained from the analysis of seed sections were consistent with those determined for ABA and OPDA standards, as were the precursor and major fragment ions observed in tandem mass spectrometry (MS/MS) imaging. Furthermore, the precursor and fragment ion images showed similar distribution patterns. In addition, the localisation of ABA and OPDA using DESI-IMS was confirmed using liquid chromatography-MS/MS (LC-MS/MS). The results indicated that ABA was mainly distributed in the radical and cotyledon of the embryo, whereas OPDA was distributed exclusively in external structures, such as the hilum and seed coat. The present study is the first to report the visualisation of plant hormones using IMS, and demonstrates that DESI-IMS is a promising technique for future plant hormone research.

Plant hormones comprise chemically diverse compounds that occur at low concentrations and regulate growth, development, and responses to external stimuli^1^. In seeds, abscisic acid (ABA) plays a key role in the induction of primary dormancy during seed development and acts as a germination repressor^2^,^3^,^4^,^5^,^6^,^7^,^8^. Recently, 12-oxo-phytodienoic acid (OPDA), a jasmonic acid (JA)-related compound, was reported to act with ABA in the regulation of seed dormancy and germination and to have a role in a proper development of an embryo^9^,^10^,^11^,^12^.

Conventional procedures, such as liquid chromatography tandem mass spectrometry (LC-MS/MS), is commonly used to analyse plant hormones^8^,^10^,^11^,^12^,^13^. However, since this requires the extraction of target compounds from plant tissues, it is difficult to investigate their distributions inside plant tissues. Therefore, a technique allowing the visualisation of ABA and OPDA inside plant tissues would be useful, but to date, one has yet to be fully established.

Imaging mass spectrometry (IMS) is a powerful tool for direct visualisation of biomolecules in biological tissues. The use of IMS for visualising small molecules in the tissues or cells of animals and plants has primarily been by matrix-assisted laser desorption ionisation (MALDI)^14^,^15^,^16^,^17^,^18^,^19^,^20^,^21^,^22^. However, the detection of small molecules using MALDI-IMS is often complicated by interfering low-mass matrix ions generating a crowded low mass region. Although ultra-high mass resolution in orbitrap^19^,^20^, MS/MS in quadrupole-time-of-flight (Q-TOF)^14^. and ion trap^17^,^21^,^22^ systems have been used to circumvent this problem, avoiding the use of the matrix altogether is desirable. Desorption electrospray ionisation (DESI) is a growing ambient ionisation technique^23^,^24^,^25^,^26^,^27^,^28^,^29^, that is initiated when charged droplets impact the biological tissues directly^23^,^24^. Unlike MALDI, DESI does not require the application of a matrix. Therefore, DESI-IMS is often considered more suitable than MALDI-IMS for the visualisation of low molecules.

The DESI ion source is commonly used with orbitrap^28^ or Q-TOF^29^ mass spectrometers. Orbitraps are useful for visualising metabolites, owing to its ultra-high mass resolution and accuracy; however, its sensitivity depends on the stability of these ions during ion cyclotron resonance. Q-TOFs have high sensitivity, mass resolution and mass accuracy in both the MS and MS/MS modes, which permits the accurate calculation of the chemical formulae for small molecules and their derived fragments.

The common bean, *Phaseolus vulgaris* L., which is one of the most important protein and micronutrient sources for both humans and livestock worldwide^30^. In 2014, the genome of *P. vulgaris* L. became available, which has provided a unique opportunity to elucidate the role of plant hormones at the molecular level. In addition, the number of genes and number of members within each gene family in *P. vulgaris* L. does not significantly differ from that of *Arabidopsis*, the most popular model plant species^30^. As a result, *P. vulgaris* L. is increasingly being used as a model legume.

The present study describes and quantifies the distributions of ABA and OPDA in immature *P. vulgaris* L. seed sections using DESI-IMS coupled to a Q-TOF mass spectrometer and LC-ESI-MS/MS analysis.

## Results and Discussion

### Preparation of immature seed sections

**Figure 1:**
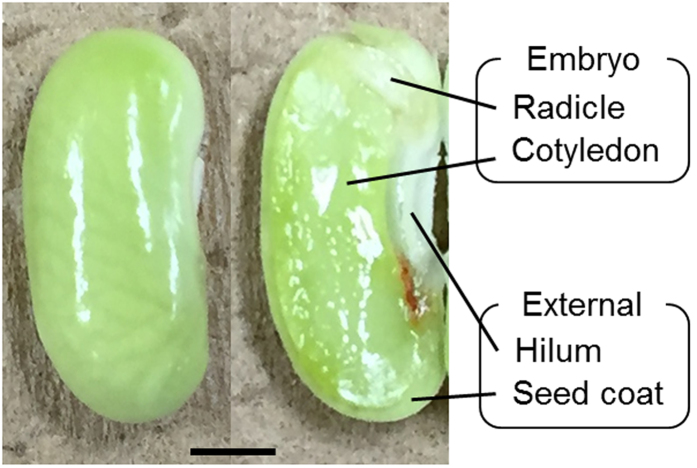
Structure of immature *Phaseolus vulgaris* L. seeds. Optical image of immature seed. Scale bar = 2 mm.

Figure 1 shows the cross-sectional structures of typical immature seeds. The ABA and OPDA contents of immature seeds are thought to change during development^8^,^10^,^12^. Using LC-ESI-MS/MS analysis, we quantified both ABA and OPDA in the immature seeds weighting between 30–210 mg. The analysis revealed that the levels of ABA and OPDA increased markedly in seeds weighting more than approximately 120 mg, and that high amounts of the ABA and OPDA were maintained in heavier seeds (data not shown). Therefore, 150– to 190-mg immature seeds were used for DESI-IMS.

**Figure 2:**
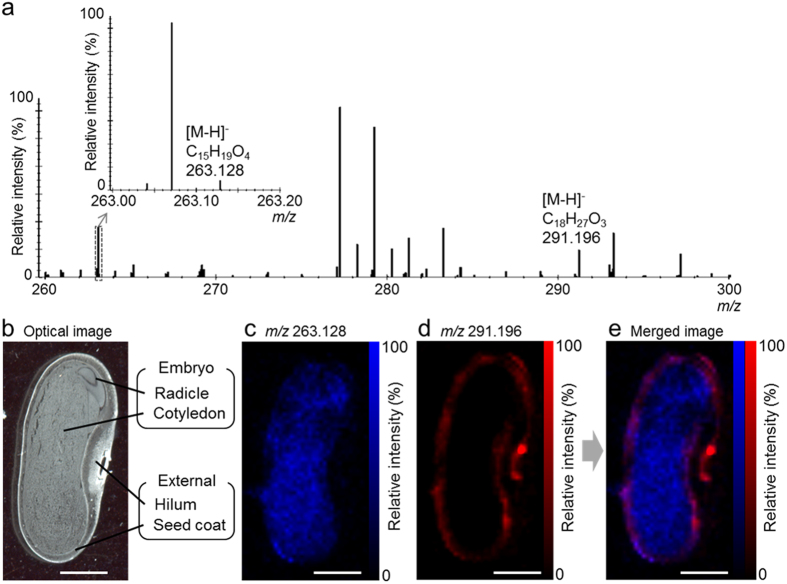
Desorption electrospray ionisation-imaging mass spectrometry (DESI-IMS) of immature *Phaseolus vulgaris* L. seed. Sections were prepared from a 163.7-mg seed. Spatial resolution was set at 200 μm. Internal mass calibration was performed post-acquisition using the exact *m/z* of the palmitic acid [M-H]^−^ ion (*m/z* 255.233). (**a**) Mass spectrum at the *m/z* 260–300. The chemical formulae are shown at the top of the respective peaks. (**b**) Optical image of a seed section after DESI-IMS. (**c**) Ion image at *m/z* 263.128. (**d**) Ion image at *m/z* 291.196. (**e**) Merged ion image of (**c** and **d**). Scale bar = 2 mm.

Because efficient sectioning is crucial for successful DESI-IMS analysis^31^, we also compared a few preparation techniques. When frozen in liquid nitrogen and sectioned at 20-μm thickness, the external structures of immature seeds, such as the hilum and seed coat, were destroyed. However, CMC freeze-embedding allowed for good quality sections of immature seeds with structures remaining intact and both the embryo and external structures clearly observed (Fig. 2b).

### DESI-IMS of immature seed sections

Using the Q-TOF, we initially measured ABA and OPDA standards using DESI-MS in the negative ion mode at 30 000 full width at half maximum (FWHM) mass resolution. Both ABA and OPDA were detected as [M-H]^−^ ions. This was followed by DESI-IMS analysis of immature seed sections (Fig. 2a). Peaks corresponding to the ABA [M-H]^−^ and OPDA [M-H]^−^ ions were detected at mass/charge (*m/z*) 263.128 and *m/z* 291.196, respectively (Table 1). The differences between the exact and detected *m/z* values were −3.4 ppm for ABA and −2.1 ppm for OPDA. The chemical formulae for the peaks were consistent with those generated from ABA and OPDA standards (Fig. 2a). The ion images at *m/z* 263.128 (Fig. 2c) and *m/z* 291.196 (Fig. 2d), together with the merged image of these ions (Fig. 2e), indicated that ABA was mainly distributed in the embryo, including the radicle and cotyledon, whereas OPDA exclusively distributed in external structures, such as the hilum and seed coat. DESI-IMS coupled to an orbitrap was also performed at 100 000 FWHM mass resolution. The OPDA [M-H]^−^ ion was detected; however, the ABA [M-H]^−^ ion was not (Supplementary Fig. S1). Therefore, the DESI-IMS orbitrap setup was not further used in this study. We hypothesized that the main reason ABA was not detected in the orbitrap because of the poor stability of the ABA ion during ion cyclotron resonance. Fortunately, the Q-TOF did not exhibit this problem, and we were also able to improve the stability and sensitivity of the ABA and OPDA signals using a new DESI sprayer design using a fixed fused silica emitter^32^.

### DESI-IMS/MS of immature seed sections

In order to further increase the specificity of the imaging experiment, we analysed the immature seed sections using DESI-IMS/MS Q-TOF^21^,^26^. The precursor and fragment ions of both ABA and OPDA were monitored.

**Figure 3:**
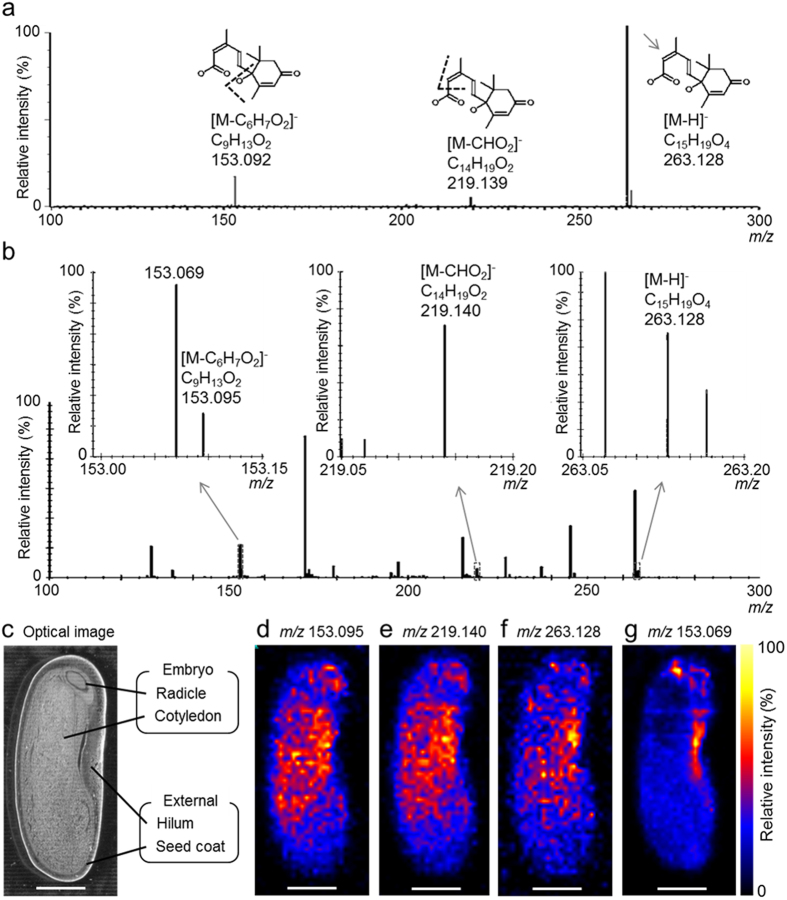
Desorption electrospray ionisation - tandem imaging mass spectrometry (DESI-IMS/MS) of abscisic acid (ABA) in immature *Phaseolus vulgaris* L. seed. Sections were prepared from a 163.7-mg seed. (**a**) Tandem mass spectrum (MS/MS) of ABA standard. The chemical formulae and structures are shown at the top of the respective peaks. (**b**) MS/MS spectrum of the immature seed section at *m/z* 262–265. Internal calibration of the MS/MS spectrum was performed post-acquisition using the exact *m/z* of ABA [M-H]^−^ ion (*m/z* 263.1289). (**c**) Optical image of seed section after DESI-IMS/MS. (**d**) Ion image at *m/z* 153.095. (**e**) Ion image at *m/z* 219.140. (**f**) Ion image at *m/z* 263.128. Scale bar = 2 mm.

DESI-MS/MS of the ABA standard precursor [M-H]^−^ ion at *m/z* 263.128 generated two major fragment ions at *m/z* 153.092 and *m/z* 219.139 (Fig. 3a). The associated chemical formulae and structures are shown on top of each peak. Subsequent DESI-IMS/MS of the immature seed sections resulted in the same precursor (*m/z* 263.128) and fragment ions (*m/z* 153.095 and *m/z* 219.140) (Fig. 3b). Another peak was observed at *m/z* 153.069 close to the ABA-derived fragment ion at *m/z* 153.095 (Fig. 3b). We found that at least 20 000 FWHM mass resolution was required to distinguish between the peaks at *m/z* 153.069 and *m/z* 153.095 (data not shown).

Images derived from the two major ABA fragment ions (Fig. 3d,e) and the ABA precursor ion (Fig. 3f) exhibited identical distribution patterns. In contrast, a different distribution pattern was observed for the *m/z* 153.069 ion (Fig. 3g). Furthermore, the ABA distribution patterns obtained using DESI-IMS/MS (Fig. 3d–f) were similar to those obtained using DESI-IMS (Fig. 2c), thereby confirming the precise localization of ABA in immature *P. vulgaris* L. seeds.

**Figure 4:**
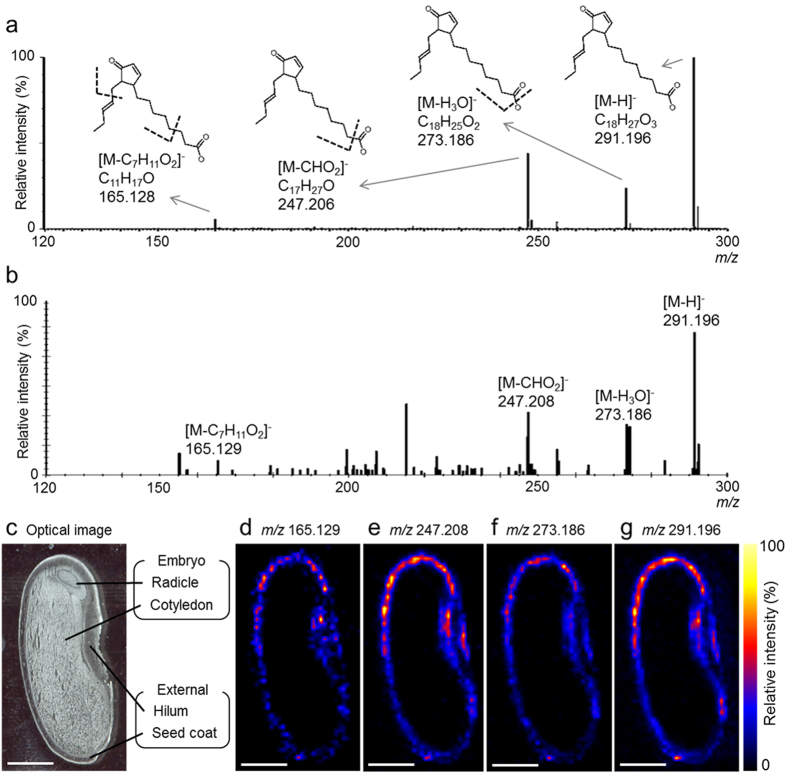
Desorption electrospray ionisation-tandem imaging mass spectrometry (DESI-IMS/MS) of 12-oxo-phytodienoic acid (OPDA) in immature *Phaseolus vulgaris* L. seed. Sections were prepared from a 163.7-mg seed. (**a**) Tandem mass spectrum (MS/MS) of the OPDA standard. The chemical formulae and structures are shown at the top of the respective peaks. (**b**) MS/MS spectrum of the immature seed section at *m/z* 290–293. Internal calibration of the MS/MS spectrum was performed post-acquisition using the exact *m/z* of OPDA [M-H]^−^ ion (*m/z* 291.1966). (**c**) Optical image of a seed section after DESI-IMS/MS. (**d**) Ion image at *m/z* 165.129. (**e**) Ion image at *m/z* 247.208. (**f**) Ion image at *m/z* 273.186. (**g**) Ion image at *m/z* 291.196. Scale bar = 2 mm.

Meanwhile, the DESI-MS/MS of the OPDA standard precursor [M-H]^−^ ion at *m/z* 291.196 resulted in three fragment ions at *m/z* 165.128, *m/z* 247.206, and *m/z* 273.186 (Fig. 4a). The associated chemical formulae and structures are shown on the top of each peak. Subsequent DESI-IMS/MS of immature seed sections resulted in the same precursor ion (*m/z* 291.196) and major fragment ions (*m/z* 165.129, *m/z* 247.208, and *m/z* 273.186; Fig. 4b). The distribution patterns obtained using DESI-IMS/MS for the OPDA precursor ion (Fig. 4g) and the three major OPDA fragment ions (Fig. 4d–f) were similar to those obtained using DESI-IMS (Fig. 2d), thereby confirming the precise localization of OPDA in immature *P. vulgaris* L. seeds.

### LC-ESI-MS/MS of ABA and OPDA in immature seeds

In order to confirm the ABA and OPDA distribution patterns observed in the DESI-IMS analysis, ABA and OPDA were quantified in the 80% methanol extracts of the embryo and external structures of four different immature seeds using LC-ESI-MS/MS (Table 2). The mean concentration of ABA in the embryo (1844 ± 478 pg/mg fresh weight (FW)) was approximately 18 times higher than that of the external structures (103 ± 52 pg/mg FW). In contrast, the mean concentration of OPDA in the external structures (8164 ± 1549 pg/mg FW) was approximately 154 times higher than that in the embryos (53 ± 24 pg/mg FW). These results agreed with those of the DESI-IMS analysis.

Goetz *et al*.^12^ reported that, in tomato, OPDA occurs almost exclusively in the seed coat tissues. They further reported that the female-sterile phenotype of the JA-insensitive mutants of tomato (*jai1*) could be rescued by the wound-induced synthesis of OPDA, which suggests that OPDA, rather than JA, is involved in embryo development. Similarly, the present study revealed that OPDA is localised in the seed coat of *P. vulgaris* L.

Meanwhile, using *Arabidopsis* mutants disrupted in peroxisomal β-oxidation, Dave *et al*.^9^,^10^ demonstrated that OPDA, rather than JA or JA-isoleucine, is involved in the repression of seed germination and that its action is synergistic with ABA. Dave *et al*.^11^ showed that the synergistic action observed in *Arabidopsis* results from the accumulation of OPDA in response to ABA and a dormancy-promoting factor, MOTHER-OF-FT-AND-TFL1. In addition to its inhibitive roles in seed germination, ABA is important in the regulation of embryo growth^7^. Therefore, ABA and OPDA constitute indispensable counterparts in the regulation of seed growth and germination. However, there is limited reliable information on the distribution of ABA in seeds. To the best of our knowledge, the present work using DESI-IMS Q-TOF is the first to report that ABA is mainly localized in the embryo including the radicle and cotyledon. In addition, the DESI-IMS Q-TOF could simultaneously visualise the distribution of ABA and OPDA in seed tissues. Furthermore, DESI-IMS had a great advantage over conventional procedures, such as LC-MS/MS, in that it can directly visualize with finer spatial precision the gradients of the ABA and OPDA concentrations inside plant tissues (Figs 2c–e, 3d–g and 4d–g).

In conclusion, it was found that the DESI-IMS is a valuable tool for the visualisation of plant hormones and investigations of their biological roles.

## Methods

### Reagents

*cis*-12-Oxo-phytodienoic acid (OPDA) was purchased from Cayman Chemical Company (Ann Arbor, MI, USA). (+)-Abscisic acid (ABA), methanol, and water were purchased from Sigma Aldrich (St. Louis, MO, USA). D_6_-ABA was purchased from Icon Isotopes (Summit, NJ, USA). D_5_-OPDA was purchased from OlChemlm Ltd (Olomouc, Czech Republic). Carboxymethyl cellulose (CMC) sodium salt was purchased from Wako Chemicals (Tokyo, Japan). Superfrost Plus microscope glass slides were purchased from Thermo Scientific (San José, CA, USA). All reagents and solvents used in the present study were of analytical grade.

### Seeds

*Phaseolus vulgaris* L. were purchased from a local supermarket, and immature seeds were removed from the pods, weighed, flash frozen in liquid nitrogen, and preserved at −80 °C until used.

### Sample preparation for LC–electrospray ionisation (ESI)–MS/MS analysis

Frozen immature seeds weighting 30–210 mg were homogenized using a disposable homogenizer, and 1 ml cold aqueous methanol (80%) was immediately added. After mixing for 30 min, the suspensions were centrifuged at 3 000 rpm for 10 min. After adding of D_6_-ABA and D_5_-OPDA in the supernatants (final concentrations were 100 ng/mL) as internal standards, the solutions were subsequently analysed using LC-ESI-MS/MS.

For the quantification of ABA and OPDA in the embryo and external structures of the immature seeds, tissues were separated from four different seeds weighting 150–190 mg, weighed, and then subjected to the same extraction procedure.

### LC–ESI–MS/MS analysis

One μl of each tissue extract was injected into an Agilent 1200 high performance liquid chromatography system coupled to an Agilent 6430 triple quadrupole mass spectrometer equipped with an ESI source (all Agilent Technologies, Palo Alto, CA, USA). An Agilent ZORBAX Eclipse XDB-C18 reversed-phase column (50 × 2.1 mm, 1.8 μm particle size; Agilent Technologies) was used. The solvents used were water (solvent A) and acetonitrile (solvent B), each containing 0.05% acetic acid. The flow rate and LC gradient conditions were 3%–70% B from 0–22 min at a flow rate of 0.2 ml/min, 98% B from 22–27 min at a flow rate of 0.3 ml/min, and 3% B from 27–37 min at a flow rate of 0.2 ml/min.

The MS instrument was operated in the negative ion mode using a drying gas temperature of 300 °C (5.0 l/min). ABA, D_6_-ABA, OPDA, and D_5_-OPDA were monitored using multi-reaction monitoring (MRM) transitions at *m/z* 263.1 > 153.1, *m/z* 269.1 > 159.1, *m/z* 291.1 > 165.1, and *m/z* 296.2 > 170.1 respectively. The collision energy (CE) was optimized for these MRM transitions (CE = 2 for ABA and D_6_-ABA, and CE = 16 for OPDA and D_5_-OPDA). D_6_-ABA and D_5_-OPDA were used as internal standards for quantification. Both Q1 (MS1) and Q3 (MS2) were maintained at 0.7 *m/z* FWHM during the LC-ESI-MS/MS experiments. Data analysis was performed using the MassHunter software (Agilent Technologies). The concentrations of ABA and OPDA in the embryo and external structures of four different immature seeds were calculated per mg of fresh weight (FW). Each value is represented by the mean ± standard deviation (n = 4).

### DESI-MS/MS analysis

One μl of aqueous methanol (50%) containing 1 ng/μl of the ABA or OPDA standards were placed on a glass slide, and allowed to dry. The MS/MS spectrum of each compound was measured using a Xevo G2-XS Q-TOF instrument equipped with a 2D DESI ion source (all Waters, Milford MA, USA). The DESI spray solvent (95% methanol:5% water, v/v) was delivered at a flow rate of 3 μl/min using a Harvard syringe pump. A voltage of 3 kV was applied to the DESI sprayer, and nitrogen was delivered at 0.4 MPa from an external gas cylinder. The optimised CE voltages for the generation of major fragment ions were 10 eV for ABA and 20 eV for OPDA. External calibration was performed by 10 mmol/L sodium formate solution in 90% 2-propanol (v/v) prior to measurement. The chemical formulae and structures of the major fragment ions were calculated using the MassFragment software (Waters).

### Preparation of seed sections for DESI-IMS

Seed sections were prepared from a 163.7-mg seed using CMC freeze-embedding^31^. Briefly, the immature seed was immersed in 2% CMC and frozen in liquid nitrogen. Subsequently, 20-μm sections were consecutively prepared using a CM 1860 cryostat (Leica Microsystems, Wetzlar, Germany). The sections were then mounted onto Superfrost microscope glass slides (Thermo Scientific), which were dried in a vacuum desiccator for 10 min. The sections were placed in 15-ml centrifuge tubes that contained silica gel for drying, and were preserved at −80 °C until DESI-IMS analysis.

### DESI-IMS analysis

DESI-IMS was performed using a Xevo G2-XS Q-TOF instrument equipped with a 2D DESI ion source (Waters). The DESI solvent (95% methanol, v/v) was delivered at a flow rate of 3 μl/min using a Harvard syringe pump. A voltage of 3 kV was applied to the DESI sprayer, and nitrogen was delivered at 0.4 MPa from an external gas cylinder. The seed sections were scanned in horizontal lines at 200 μm/s, and collecting a mass spectrum every second. Scanning rows were separated by vertical steps of 200 μm, resulting in a lateral resolution of 200 μm. Signals at *m/z* 200–400 were measured in the negative ion mode at 30 000 FWHM mass resolution. External calibration was performed by 10 mmol/L sodium formate solution in 90% 2-propanol (v/v) prior to measurement. Internal calibration was performed post-acquisition in the HDImaging software (Waters) using the exact *m/z* of the palmitic acid [M-H]^−^ ion (*m*/z 255.233). Peak picking was performed in order of the most intense peaks from the entire *m/z* range at 30 000 FWHM mass resolution, and ion images were constructed using the HDImaging software (Waters). Chemical formulae and structures were calculated using the MassFragment and Elemental Composition software (Waters).

DESI-tandem imaging mass spectrometry (IMS/MS) was performed at *m/z* 262–265 for ABA and *m/z* 290–293 for OPDA, respectively. The CE was set at 10 eV for ABA and 20 eV for OPDA. Internal calibration was performed post-acquisition in the HDImaging software (Waters) using the exact *m/z* of the precursor [M-H]^−^ ions (*m/z* 263.1289 for ABA and *m/z* 291.1966 for OPDA). The major fragment ions from ABA and OPDA were used to construct ion images using the HDImaging software (Waters). The chemical formulae and structures were calculated using the MassFragment and Elemental Composition software (Waters).

Ultra-high mass resolution imaging was also performed using the Exactive (Thermo Scientific) orbitrap mass spectrometer equipped with a 2D DESI ion source (Prosolia Inc. Indianapolis, IN, USA). Methanol (95%) was used as the spray solvent. Spatial resolution was set to 150 μm, and mass resolution was set at 100 000 FWHM (@*m/z* 400). Signals at *m/z* 200–400 were measured in the negative ion mode. Image files (IMG files) were created from the acquired data using the FireFly software, and processed to generate images using the BioMAP software (Novartis, Basel, Switzerland). Chemical formulae were calculated using the Xcalibur software (Thermo Scientific).

### Statistical analysis

The concentrations of ABA and OPDA in the embryo and external structures are the means of four different seeds ± standard deviation. Statistical differences between the means were evaluated using Student’s *t*-test (*P* < 0.05).

## Additional Information

**How to cite this article**: Enomoto, H. *et al*. Visualisation of abscisic acid and 12-oxo-phytodienoic acid in immature *Phaseolus vulgaris* L. seeds using desorption electrospray ionisation-imaging mass spectrometry. *Sci. Rep.* **7**, 42977; doi: 10.1038/srep42977 (2017).

**Publisher's note:** Springer Nature remains neutral with regard to jurisdictional claims in published maps and institutional affiliations.

## Supplementary Material

Supplementary Information

## Figures and Tables

**Table 1:** Desorption electrospray ionisation-imaging mass spectrometry (DESI-IMS) of abscisic acid (ABA) and 12-oxo-phytodienoic acid (OPDA).

Analyte	Chemical Formula	Exact Mass (Da)	Exact *m/z* [M-H]^−^	Detected *m/z* [M-H]^−^	Error (ppm)
ABA	C_15_H_20_O_4_	264.1362	263.1289	263.128	−3.4
OPDA	C_18_H_28_O_3_	292.2038	291.1966	291.196	−2.1

**Table 2:** Abscisic acid (ABA) and 12-oxo-phytodienoic acid (OPDA) concentrations in the embryo and external structures of immature *Phaseolus vulgaris* L. seeds.

Tissue	Weight (mg FW)	OPDA (pg/mg FW)	ABA (pg/mg FW)
Embryo	113 ± 7	53 ± 24	1844 ± 478
External structures	59 ± 1	8164 ± 1549	103 ± 52

ABA and OPDA in aqueous methanol (80%) extracts of embryo and external structures separated from four different immature seeds were quantified by liquid chromatography-electrospray ionisation-tandem mass spectrometry (LC-ESI-MS/MS) analysis. Concentrations of ABA and OPDA in embryo and external structures are shown per mg of fresh weight (FW). Each value is represented by the mean ± standard deviation (n = 4).
