# Supplementary material for: Visualisation of abscisic acid and 12-oxo-phytodienoic acid in immature Phaseolus vulgaris L. seeds using desorption electrospray ionisation-imaging mass spectrometry
Source: Sci Rep. 2017 Feb 17;7:42977. doi: 10.1038/srep42977 (PMC5314351; doi:10.1038/srep42977)
Supplement: Supplementary Information [file srep42977-s1.doc]

**SUPPLEMENTARY INFORMATION**

**Visualisation of abscisic acid and 12-oxo-phytodienoic acid in immature *Phaseolus vulgaris* L. seeds using desorption electrospray ionisation-imaging mass spectrometry**

Hirofumi Enomoto^1,2,*^, Takuya Sensu^1^, Kei Sato^2^, Futoshi Sato^3^, Thanai Paxton^3^, Emi Yumoto^1^, Koji Miyamoto^1^, Masashi Asahina^1,2^, Takao Yokota^1^ & Hisakazu Yamane^1,2^

^1^Department of Biosciences, Faculty of Science and Engineering, Teikyo University, Utsunomiya 320-8551, Japan.

^2^Division of Integrated Science and Engineering, Graduate School of Science and Engineering, Teikyo University, Utsunomiya 320-8551, Japan.

^3^Waters Corporation, Shinagawa 140-0001, Japan.

**^*^**Correspondence and requests for materials should be addressed to H.E. (email: enomoto@nasu.bio.teikyo-u.ac.jp)

**
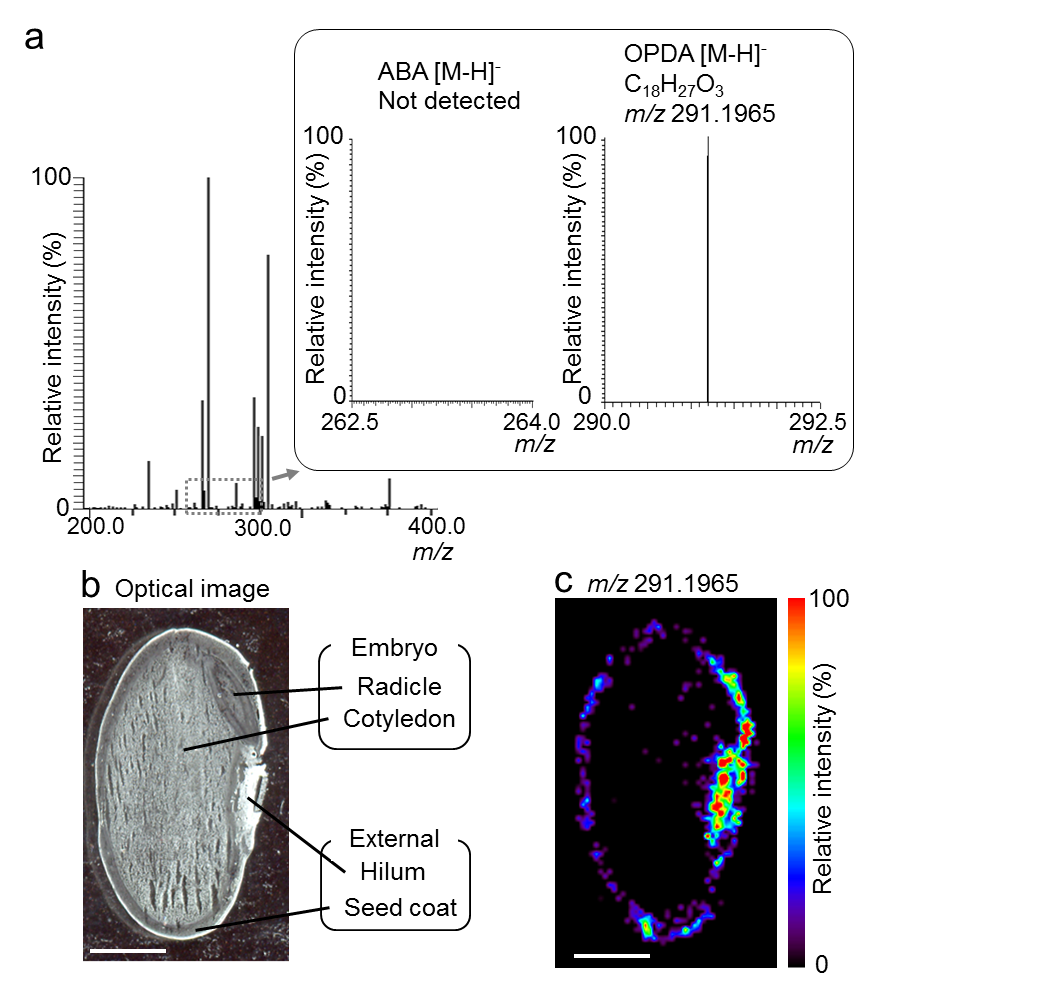
**

**Supplementary Figure S1. Desorption electrospray ionization-imaging mass spectrometry (DESI–IMS) of immature seed sections using an orbitrap mass spectrometer.** Seed sections were prepared from a seed weighing 174.1 mg. Mass and spatial resolutions were set at 100 000 FWHM and 150 µm, respectively. Aqueous methanol (95%) was used as a spray solvent. (**A**) Mass spectrum at the *m/z* 200−400. The chemical formula is shown at the top of the respective peak. (**B**) Optical image of the immature seed section after DESI–IMS. (**C**) Ion image at *m/z* 291.1965. Scale bar = 2 mm.
